# PROMRIINE (PRe-operatory Magnetic Resonance Imaging is INEffective) Study: A Systematic Review and Meta-analysis of the Impact of Magnetic Resonance Imaging on Surgical Decisions and Clinical Outcomes in Women with Breast Cancer

**DOI:** 10.1245/s10434-024-15833-5

**Published:** 2024-07-27

**Authors:** André Mattar, Marcelo Antonini, Andressa Amorim, Evandro Falaci Mateus, Fabio Bagnoli, Francisco Pimentel Cavalcante, Guilherme Novita, Lincon Jo Mori, Marcelo Madeira, Marina Diógenes, Antônio Luiz Frasson, Eduardo de Camargo Millen, Fabrício Palermo Brenelli, Lucas Miyake Okumura, Felipe Zerwes

**Affiliations:** 1 Grupo Oncoclínicas-SP, São Paulo, SP Brazil; 2 Hospital da Mulher- SP, São Paulo, SP Brazil; 3 Hospital do Servidor Público Estadual – Francisco Morato de Oliveira, São Paulo, SP Brazil; 4 Instituto de Pesquisa Prevent Senior, São Paulo, SP Brazil; 5 Faculdade de Ciências Médicas da Santa Casa de São Paulo, São Paulo, SP Brazil; 6 Hospital Geral de Fortaleza, Fortaleza, CE Brazil; 7 Hospital Sírio Libanês, São Paulo, SP Brazil; 8 Faculdade Israelita de Ciências da Saúde Albert Einstein, São Paulo, SP Brazil; 9 Hospital Israelita Albert Einstein, São Paulo, SP Brazil; 10 Americas Oncologia, Rio de Janeiro, RJ Brazil; 11 Value ArchTech, São Paulo, SP Brazil; 12 Universidade Estadual de Campinas, Campinas, SP Brazil; 13 Pontificia Universidade Católica do Rio Grande do Sul, São Paulo, RS Brazil

**Keywords:** Breast cancer, Magnetic resonance imaging, Systematic review, Meta-analyses, Randomized controlled trial

## Abstract

**Background:**

The purpose of this study was to review and summarize the association between preoperative magnetic resonance imaging (MRI) and surgical outcomes in women with newly diagnosed invasive breast cancer from published randomized controlled trials (RCT).

**Materials and Methods:**

Two independent researchers conducted a systematic review through a comprehensive search of electronic databases, including PubMed, Medline, Embase, Ovid, Cochrane Library, and Web of Science. If there was disagreement between the two reviewers, a third reviewer assessed the manuscript to determine whether it should be included for data extraction. The quality of the papers was assessed using the risk of bias tool, and the evidence was analyzed using GRADE. Meta-analyses using a fixed-effects model were used to estimate the pooled risk ratio (RR) and 95% confidence interval (CI).

**Results:**

Initially, 21 studies were identified, 15 of which were observational comparative studies. A total of five RCTs were included, and they suggested that preoperative MRI significantly reduced the rate of immediate breast-conserving surgery and increased the risk for mastectomy.

**Conclusions:**

From the RCT perspective, preoperative MRI for newly diagnosed invasive breast cancer did not improve surgical outcomes and may increase the risk of mastectomy.

Breast-conserving surgery (BCS) is the standard treatment for early stage breast cancer (BC),^1,2^ and no ink on the tumor is considered as clear margins^3^ for invasive BC. Therefore, preoperative evaluation is essential once multifocality and multicentricity are well-known characteristics of BC.^4^ Magnetic resonance imaging (MRI) is not only a morphological examination, but also a functional technique that provides a higher detection rate of additional lesions than mammography and breast ultrasound.^5^ MRI is typically used for BC staging.^6,7^ However, according to a meta-analysis study, the incremental cancer detection rate was only 4.2% [95% confidence interval (CI) 2.7–6]; its false-positive rates are still considered high, leading to unnecessary biopsies and eventually surgical procedures that result in benign pathological results.^8–10^

Currently, BC specialists recommend that MRI may be useful for some groups of patients with BC, such as those with: (1) invasive lobular carcinoma;^11,12^ (2) aggressive types of BC, such as triple-negative and human epidermal growth factor receptor 2-positive tumors;^13^ (3) dense breast tissue or younger age, especially age < 50 years;^14^ and (4) those who plan to undergo neoadjuvant treatment.^15,16^ MRI is also used for BC screening, especially in patients with *BRCA-*mutated BC^17–19^ and those with BC with metastatic lymph node disease with occult primary;^20^ however, it is not yet considered a standard of care^8–10^ for preoperative purposes.

In this context, in 2023,^21^ a newly published randomized trial composed a list of evidence that assessed the impact of MRI in planning BC surgeries.^21^ The trial conducted by the research group included surgical management, local and systemic recurrences, and survival as important outcomes. Therefore, considering the current evidence, a new systematic review with meta-analyses is required to assess whether a new trial can change previous estimates.

To summarize, we hypothesized that women exposed to MRI might have detrimental outcomes due to a higher risk of extensive surgeries, lower quality of life, and no proven clinical benefits such as survival, disease-free survival, and other cancer-related outcomes. Therefore, the main objective of this study was to assess the effectiveness of MRI as a preoperative tool in women with BC.

## Materials and Methods

### Protocol Registration and Rationale of Review

PROMRIINE is a systematic review registered at PROSPERO/University York (CRD42023422415).

This study sought to identify the randomized controlled trials (RCTs) that assessed whether MRI exposure in women with BC was associated with higher levels of surgical procedures such as initial mastectomy and reoperation. Therefore, our research question was as follows: “In RCT, what was the clinical impact (surgery-related outcomes and clinical outcomes) of the use of magnetic resonance imaging (MRI) in women with breast cancer?”

### Data Sources and Searches

Five databases were screened for studies: PubMed/MEDLINE, Scopus, Cochrane Central Register of Controlled Trials, and Google Scholar (manual search). The searches included manuscripts published between January 2012 and July 2023 with no language restrictions.

### Study Selection

Two independent reviewers screened the database on the basis of the title and abstract and included papers that demonstrated adherence to the research strategy defined in the systematic review protocol. If there was disagreement between the two reviewers, a third reviewer assessed the manuscript to determine whether it should be included for data extraction.

### Data Extraction

Two independent reviewers extracted the following data (in duplicate) from all included studies: author, year of publication, number of study sites, study design, baseline population information (age), BC staging, receptor status, tumor localization, number of BCSs and mastectomies as initial procedures, the need for reoperation, recurrence, and death. A third reviewer was also available to solve any discrepancies.

### Outcomes, Data Synthesis, and Analysis

The main outcomes considered in this review were (1) number of BCSs, (2) number of initial mastectomies, (3) need for reoperation, and (4) need for additional mastectomy. The exploratory outcomes included BC recurrence and death.

The studies are tabulated (Tables 1 and 2). The pooled relative risks were calculated on the basis of the number of events described in each study. Meta-analyses were conducted using R software with a fixed effects model. Heterogeneity was assessed using the *I*^2^ method. RCTs were also assessed for the risk of bias, which is available in an MS Excel-based form at https://www.riskofbias.info, and the quality of the evidence was assessed using the Grading of Recommendations Assessment, Development and Evaluation GRADE (freely available at http://www.gradeworkinggroup.org). Overall, the risk-of-bias method relies on assessing the quality of randomization, deviations from the initial intervention plan, missing data on outcomes, or selective reporting. GRADE assesses the quality of the evidence by rating the risk of bias and (in)consistency of the summarized outcome from the study and others.

**Table 1:** RCT characteristics

Author, year	Country	Multicentric study	Brief description of the study	Sample MRI	Sample non-MRI
Mota et al.^34^	Brazil	No	BREAST-MRI is a randomized, open label, unblinded trial	255	267
Peters et al.^37^	Netherlands	No	MONET study was a RCT designed to assess patients with a nonpalpable BI-RADS 3–5 lesion. Patients were randomly assigned to receive routine medical care, including mammography, ultrasound, and lesion sampling by large core needle biopsy or additional MRI preceding biopsy.	207	211
Bruck et al.^35^	Finland	No	Randomized clinical study	50	50
Gonzalez et al.^38^	Sweden	Yes	The POMB trial (Preoperative MRI of the Breast) assessed the role of preoperative MRI in BC	220	220
Turnbull et al.^36^	UK	Yes	Open randomized, parallel group trial in 45 UK centers, mostly with women with invasive carcinoma.	817	808

**Table 2:** Clinical information from women enrolled in the studies

	Sample		Age (means or medians)		Post-menopausal status		DCIS		HER2+ receptor		Progesterone-positive receptor		Estrogen-positive receptor		Stage (0, I, II)	
	MRI	Non-MRI	MRI	Non-MRI	MRI	Non-MRI	MRI	Non-MRI	MRI	Non-MRI	MRI	Non-MRI	MRI	Non-MRI	MRI	Non-MRI
Mota et al.^34^	255	267	56.9	57.1	167 (65%)	185 (69%)	–	–	6 (2%)	7 (2%)	–	–	–	–	252 (99%)	267 (100%)
Peters et al.^37^	207	211	55	56	–	–	41 (20%)	41 (19%)	–	–	–	–	–	–	–	–
Bruck et al.^35^	50	50	61	61	–	–	–	–	6 (12%)	8 (16%)	46 (92%)	41 (82%)	46 (92%)	44 (88%)	–	–
Gonzalez et al.^38^	220	220	46	46	10 (4%)	17 (8%)	–	–	–	–	–	–	–	–	–	–
Turnbull et al.^36^	817	808	57	57	574 (70%)	565 (70%)	–	–	–	–	–	–	–	–	–	–

### Ethics Approval

Informed consent and ethics approval were not required as this systematic review was based on published studies.

## Results

**Fig. 1:**
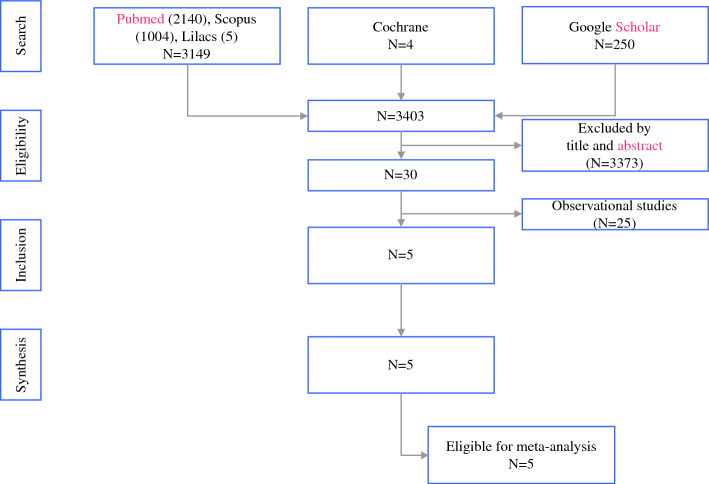
Study selection process

Of the 3403 initially identified studies, 3373 did not have titles or abstracts aligned with the inclusion criteria. After reading 30 studies, the reviewers agreed to include 5 RCTs (Fig. 1).

This review included 3870 randomized women with BC, of whom 1856 underwent MRI and 2014 did not (non-MRI group). The study population comprised women aged 46–61 years with stage I or II BC. When specified, most studies reported women on post-menopausal status (65% of the sample). One study reported data on the localization of the tumor, where approximately 20% of the sample had in situ ductal carcinoma, and two studies reported information on receptor positivity. Mota et al.^21^ included 13 patients with positive statuses of human epidermal growth factor receptor 2, and Bruck et al.^22^ included women with hormone receptor-positive BC (Tables 1 and 2).

### Initial Surgery: BCS and Mastectomy

**Fig. 2:**
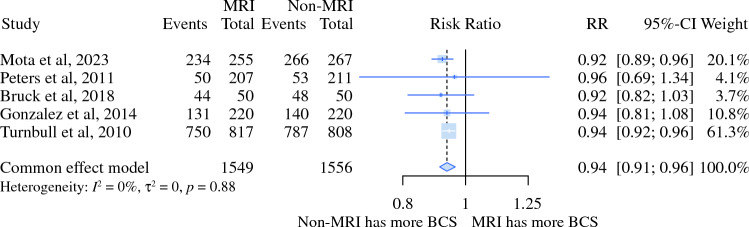
Relative risk meta-analysis for initial BCS

**Fig. 3:**
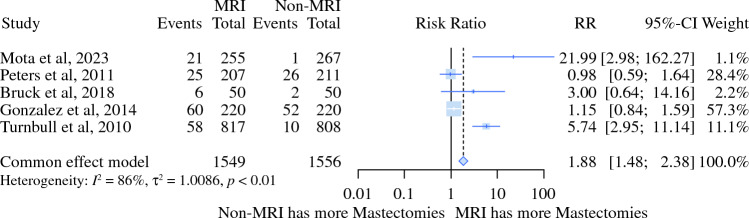
Relative risk meta-analysis for initial mastectomy

MRI was associated with 5% lower rates of BCS [risk ratio (RR) 0.95, 95% CI 0.92–0.97) and increased rates of initial mastectomy (RR 1.59, 95% CI 1.33–1.92). This evidence indicated moderate and high levels of certainty on the basis of the GRADE tool (Figs. 2 and 3; Table 3).

**Table 3:** GRADE evidence profile

Certainty assessment			Patients per group		Effect		Importance
Number of studies	Risk of bias	Certainty	MRI (*n*/*N*)	Non-MRI (*n*/*N*)	RR (95% CI)	Absolute risk (95% CI)	
Breast-conserving surgery as initial procedure							
5	Not serious	Moderate α	1456/1856	1670/2014	0.95 (0.92, 0.97)	5% decrease (2–7%) in BCS with MRI compared with non-MRI	Critical
Mastectomy as initial procedure							
5	Not serious	High	233/1856	170/2014	1.59 (1.33, 1.92)	4% increase (2–7%) in initial mastectomy with MRI	Critical
Reoperation							
5	Not serious	Low β	172/1856	212/2014	0.87 (0.72, 1.05)	No statistical difference.	Critical
New mastectomy							
5	Not serious	Low β	87/1856	107/2014	0.87 (0.66, 1.14)	No statistical difference.	Critical
Local recurrence							
1	Not serious	Low £	2/255	3/267	0.65 (0.11, 3.89)	No statistical difference.	Critical
Distant recurrence							
1	Not serious	Low £	4/255	5/267	0.83 (0.23, 3.08)	No statistical difference.	Critical
Death							
1	Not serious	Low £	12/255	10/267	1.25 (0.55, 2.86)	No statistical difference.	Critical

Observations: Risk of bias was assessed through risk of bias (RoB v.2) from Cochrane. This table is an adaptation of GRADE reporting system, considering risk of bias, indirectness, imprecision, and other features

Legends: £ (Certainty was considered low due to only 1 report in the literature and imprecision); α (conservative breast surgery as initial procedure, reoperation and new mastectomy were downgraded due to imprecision); β (evidence was downgraded because there is no statistical difference between groups (MRI versus non-MRI for reoperation and new mastectomy)

### Further Need for Surgery: Any Reoperation and Mastectomy

**Fig. 4:**
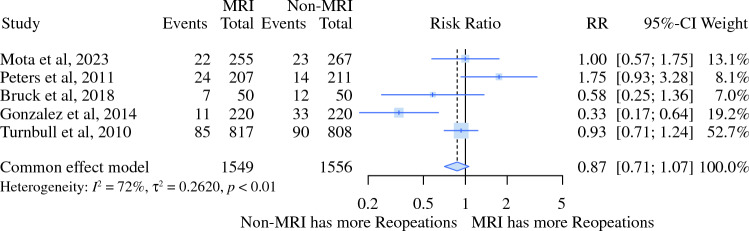
Relative risk meta-analysis for further procedure (reoperation)

**Fig. 5:**
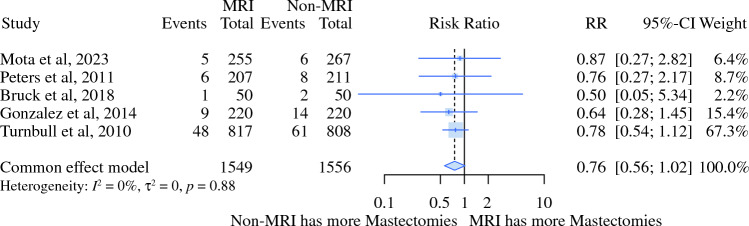
Relative risk meta-analysis for mastectomy after initial BCS

Using MRI was not statistically different (5% alpha) from the non-MRI group based on the outcomes, such as the need for further reoperation (RR 0.87, 95% CI 0.72–1.05) and additional mastectomies (RR 0.87, 95% CI 0.11–3.89) (Figs. 4 and 5; Table 3).

### Recurrence and Death

Only Motta et al.^21^ reported data on recurrence and death. In this study, MRI use was not considered statistically different from MRI non-use (non-MRI group) (5% alpha) in women with BC with one local recurrence (RR 0.65, 95% CI 0.11–3.89), distant recurrence (RR 0.83, 95% CI 0.23–3.08), or death (RR 1.25, 95% CI 0.55–2.86), as presented in Table 3.

### Quality of the Studies and Evidence

Overall, the evidence on BCS as the initial procedure was considered to have moderate certainty due to imprecision in the meta-analysis; however, the outcome was considered critical, and the risk of bias was considered low (not serious). The evidence on mastectomy as the initial procedure as the outcome was considered a “high level of certainty.” As presented in Table 3, reoperation, new mastectomy, recurrence, and death were all considered low certainty because of the lack of statistical significance. Importantly, no study was downgraded due to a lack of blinding because MRI cannot be masked as part of the intervention.

## Discussion

The PROMRIINE study is the first systematic review and meta-analysis to include only RCTs that have analyzed the outcomes of MRI in women with BC. The evidence was also assessed through the risk of bias scale and GRADE. Five RCTs were included in our analyses, which are summarized below.

The COMICE Trial^23^ was a prospective trial in 45 UK centers, with 1623 women. The patients were randomly assigned to undergo either MRI (*n* = 816) or no further imaging (*n* = 807). The primary endpoint was the proportion of patients undergoing a repeat operation or further mastectomy within 6 months of random assignment or a pathologically avoidable mastectomy at the initial operation. The addition of MRI to conventional triple assessment was not significantly associated with a reduced reoperation rate, with 153 (19%) requiring reoperation in the MRI group versus 156 (19%) in the non-MRI group (*p* = 0.77).

The MONET Trial^24^ RCT in the Netherlands included patients with nonpalpable Breast Imaging-Reporting and Data System (BI-RADS) 3–5 lesions. The patients were randomly assigned to receive routine medical care or additional MRI before biopsy. The primary endpoint was the rate of additional surgical procedures (re-excision or conversion to mastectomy) in patients with nonpalpable BC. A total of 418 patients were randomized: 207 were allocated to the MRI group and 211 to the control group. The primary BCS rate was similar in both groups; however, the number of re-excisions was paradoxically increased in the MRI group 18/53 (34%) versus 6/50 (12%) in the control group (*p* = 0.008). The number of conversions to mastectomy did not differ significantly between groups.

The POMB Trial^25^ was a prospective, randomized, multicenter study that included 440 patients aged < 56 years with BC from three Swedish breast units. Patients were randomly allocated (1:1) to either the preoperative staging with breast MRI (*n* = 220) or no breast MRI (*n* = 220) group. In patients randomized to the MRI group, a change from suggested breast conservation to mastectomy occurred in 23 of 153 (15%) patients. Breast MRI provided additional information in 83 of 220 (38%) patients, resulting in a change in treatment plan in 40 (18%). The breast reoperation rate was significantly lower in the MRI group: 11 of 220 (5%) versus 33 of 220 (15%) in the control group (*p* < 0.001). The number of mastectomies, axillary reoperations, and the number of patients receiving neoadjuvant chemotherapy after definitive treatment did not differ significantly between the groups.

Brück et al.^22^ published an RCT in 2018 from Finland investigating the diagnostic value of preoperative MRI and its impact on short-term surgical outcomes in patients with newly diagnosed unifocal stage I invasive ductal carcinoma. A total of 100 patients were randomized (1:1) to receive either preoperative MRI or to be scheduled directly for surgery. There were 50 patients in both study arms. MRI detected an additional finding in 28%, and in 7%, it was identified as malignant, causing a change in planned surgical management in 10 patients (20%). Mastectomy and reoperation rates were similar in both groups. The mean interval between referral and first surgical procedure was 34 days in the MRI group and 21 days in the control group (*p* < 0.001).

The BREAST-MRI Trial^21^ was a phase III, randomized, open-label, single-center Brazilian trial that included female participants with stage 0–III BC who were eligible for BCS. This study compared the role of MRI in preoperative evaluation with that of routine radiologic examinations (mammography and ultrasound) in BCS candidates. The primary outcome was local relapse-free survival, and secondary outcomes were overall survival, mastectomy, and reoperation rates. The study randomized 524 patients to the preoperative MRI group (*n* = 257) or control group (*n* = 267); however, the preoperative MRI increased the mastectomy rates by 8%, and the use of preoperative MRI did not influence local relapse-free survival, overall survival, or reoperation rates.

This review revealed that MRI is associated with a 59% increased rate of initial mastectomy as the first procedure and reduced rates of BCS in comparison to those women not exposed to routine MRI.

The frequency at which mastectomy rates are reported does not necessarily indicate the efficacy of MRI in surgical decision-making processes. Several methodological limitations in the existing data compromise its interpretability, including patients already scheduled for BCS; confounding variables such as tumor characteristics or patient-specific factors are often unaccounted for; and mastectomy decisions frequently reflect factors beyond medical necessity, such as surgeon or patient preference, institutional protocols, and socioeconomic or ethnic disparities.^26^

Furthermore, some studies that reported elevated mastectomy rates associated with MRI did not rigorously validate whether newly identified lesions were benign or malignant. In some cases, histopathological examination of mastectomy specimens has suggested that alterations in the initial surgical approach are not clinically warranted.

Optimal practice guidelines recommend that any additional suspicious lesions identified via MRI be subjected to biopsy or equivalent confirmatory tests if they can potentially modify the surgical strategy. Institutions offering MRI should possess biopsy capabilities to minimize surgical delays and enhance the overall care quality. Additionally, continuously monitoring quality indicators specific to MRI performance is crucial. Proficiency in the entire MRI process, from image acquisition to interpretation, not only improves diagnostic accuracy, but also fosters innovation in the field.^27^

Most studies in favor of the use of MRI as a preoperative tool have focused on the sensitivity of the method, which is much higher than that of mammography or ultrasound,^28^ especially for small tumors.^5^ They further advocate that the identification of additional disease is important for prognosis and cure; however, we know from the studies that additional disease is common^29^ and does not seem to affect survival.^1^

Some specialists advocate that MRI should be used to reduce the risk of reoperation. In a meta-analysis conducted by Eisen et al.,^26^ which amalgamates data from both randomized and retrospective studies, a statistically significant reduction in both reoperation and recurrence rates was observed. However, the robustness of these findings diminishes when prospective and retrospective studies are combined, thereby compromising the impact of the meta-analysis.

In contrast, our study, which exclusively encompassed prospective studies, revealed no statistically significant variations in either recurrence or reoperation rates between the MRI- and non-MRI-exposed populations. Moreover, our data indicate that the utilization of MRI increases the frequency of mastectomy procedures yet does not yield any measurable improvement in recurrence rates.

Another important point would be the improved detection of lobular invasive carcinoma (LIC), both in the index lesion and in a possible foci, as we know that this histological type presents greater multicentricity and bilaterality, and MRI seems to be able to assess better this subtype.^12^ Unfortunately, none of the RCTs objectively evaluated ILC and none of them were designed to exclusively assess this subtype.

This scientific evidence showed consistent results across the five RCTs and suggests that additional trials should not provide different information from what is already known in terms of surgical outcomes.

It is important to move forward and address different scientific questions. Therefore, future studies should not expose women to unnecessary research.

For example, Mota et al. (2023) was the only study demonstrating the clinical impact of using MRI in BC.^21^ During short-term follow-up (3 years), MRI did not differ from not using MRI in terms of recurrence and death risks. Many factors, such as the subtype of BC, can impact survival or disease-free survival. However, triple-negative disease can also be detected by MRI (higher sensitivity, 92–100%), as it is characterized as a larger, well-defined, and more necrotic lesion, impacting disease management.^30^ Histological characterization of BC (e.g., ductal carcinoma in situ or invasive ductal carcinoma) will play an important role in describing the included population in future studies. According to Lee et al. (2020),^31^ ductal carcinoma in situ was likely to result (*p* < 0.001) in more surgical plan changes than invasive ductal carcinoma due to the extent of suspicious lesions on breast MRI, detection of additional nodules, and other findings.

Age at menopause and the type of neoadjuvant chemotherapy are also well-characterized covariates to be assessed as they might impact prognosis.^32^ However, this information was not clearly stated in the studies included in this review.

Finally, breast density is a key factor that may affect physicians’ preference for MRI.^33^ Despite the importance of breast density, there was considerable heterogeneity in reporting the baseline characteristics of the included patients in the RCTs; some reported body mass index as a proxy for breast density, but no study has reported information on this. Therefore, this might be a relevant gap that can be addressed in future studies about the clinical impact of MRI in the BC population.

The present study has some limitations. Despite relevant findings on surgical outcomes, the amount of data on relevant clinical outcomes, such as recurrence and death, remain limited. Additionally, the absence of relevant baseline information might result in problems interpreting the evidence on neoadjuvant chemotherapies that can alter survival or even breast density, which is associated with more MRI, and possibly, more mastectomies. This study provides scientific information to inform of the benefits of using MRI on surgical outcomes; the uncertain benefits and potential harm of mastectomies should be discussed with women with BC.

This systematic review with meta-analyses provides robust evidence and a relevant discussion on the increased risks of mastectomies and reduced rates of BCS when exposing women with BC to preoperative MRI, which should be considered when offering routine practice to patients with BC before surgery. The lack of relevant baseline characteristics (breast density, age at menopause, tumor type, type of neoadjuvant chemotherapy, and others) and lack of assessment of clinical outcomes (local recurrence, systemic recurrence, event-free survival, and long-term survival) in RCTs suggest the need for further research in this field.

## Data Availability

All data are available.
